# The neural code of perceptual inference

**DOI:** 10.1186/s13041-026-01299-x

**Published:** 2026-04-01

**Authors:** Hyeyoung Shin

**Affiliations:** School of Biological Sciences, Seoul National University, Seoul, Republic of Korea

**Keywords:** Perceptual inference, Bayesian inference, Marr’s three levels, Efficiency vs. robustness tradeoff, Discrimination vs. generalization tradeoff

## Abstract

Perception is a process of inference, whereby incoming sensory evidence is interpreted based on prior expectations about the sensory world. Thus, the neural code of perception should be evaluated based on how optimally it computes perceptual inference. However, the neural code of perception has conventionally been evaluated by its capacity to represent sensory information faithfully. Due to this misalignment in the computational goal of perception, assessments of the neural code have been biased towards discriminability over generalizability, and efficiency over robustness. In this review, I suggest ways in which we can evaluate the neural code based on its capacity to achieve the goal of perception, that is, perceptual inference.

## Goal of perception: information vs. inference

When a mouse is scanning a room for food, it needs to parse every piece of sensory information to evaluate the probability that the underlying source is food, a threat, or neither. However, it does not need to encode every bit of sensory information with high fidelity. The mouse may allocate selective attention for high fidelity sensory encoding if a certain object appears to be food. Even then, probabilistic object recognition, which requires inference, must occur in tandem with high fidelity sensing.

Probabilistic perceptual inference and high-fidelity sensing are distinct goals of perception. These goals can be synergistic; accurate sensory information helps achieve better inference, and quick inference of object identity helps allocate selective attention to achieve more accurate sensing. However, it is often not necessary to do both; in the above scenario, the mouse should promptly ignore sensory information that is unlikely to be food nor a threat. Moreover, achieving these goals can involve a tradeoff under time constraints. For example, if the mouse deems a figure to be a predator, it has no time for accurate discernment of the figure’s fine features; instead, it must quickly run away.

The fundamental goal of perception is to make optimal inferences about the underlying source of sensory inputs, based on our prior knowledge of the sensory world and our internal and external context. Thus, to understand the neural code of perception, we must evaluate its capacity for perceptual inference. However, neuroscientists have often evaluated the neural code of perception based on its fidelity to sensory information, without considering its capacity for inference. For example, a topic of keen interest in the field is how trial-to-trial variability affects neural information [1–5]. These studies have shown that trial-to-trial variability can be, and often is, information limiting, especially when it is shared across neurons. Variability is inherently harmful to a code for sensory fidelity because for such computations, a fixed solution must exist for a given problem; in other words, a fixed representation is ideal for a fixed sensory input. On the contrary, the solution for a given inference problem changes based on prior expectations, encompassing internal context, external context and the subject’s knowledge base. Thus, trial-to-trial variability can be a beneficial feature for probabilistic inference, as it enables the representation of probability and uncertainty [6–10].

When information is used as the gold standard for evaluating the neural code, emphasis is put on discriminability and efficiency. This is evident in the information theory metrics used to evaluate the neural code [11, 12]: Fisher information is tied to discriminability, and Shannon information is tied to efficient coding. Relatedly, when decoding sensory stimuli from neural activity, cross-validated accuracy quantifies how well sensory representations can be discriminated, and the number of neurons needed to achieve a certain accuracy quantifies efficiency. However, a sensory representation with a given accuracy involves a tradeoff between discriminability and generalizability, as well as a tradeoff between efficiency with robustness. A more comprehensive evaluation of the neural code should assess generalizability and robustness in addition to discriminability and efficiency.

How can we assess the neural code based on its capacity to compute inference? To this end, Bayes’ theorem can be used as the gold standard for optimal inference. Bayes’ theorem states that the posterior probability is proportional to the likelihood multiplied by the prior probability:

$$\:P\left( {H|E} \right) = \:\frac{{P\left( {E|H} \right) \cdot P\left( H \right)}}{{P\left( E \right)}}$$

Here, the posterior P(*H*|*E*) describes the probability that hypothesis *H* is true given evidence *E*; the likelihood P(*E*|*H*) describes the probability of observing evidence *E* assuming hypothesis *H* is true; the prior P(*H*) describes the probability that hypothesis *H* is true before any evidence is observed; and the marginal likelihood P(E) is a normalization factor that accounts for the total probability of the evidence under all possible hypotheses.

When perception is cast as a Bayesian inference problem, the evidence is given by the sensory input, and the hypothesis refers to the brain’s interpretation of the sensory input. The prior is the hypothesis space of all plausible interpretations, and the posterior corresponds to the percept. Note, the prior is broadly defined as expectations about the sensory input, formulated by the subject’s knowledge of the sensory world and modulated by their context. For example, a professional cook may be better at identifying individual ingredients in a complex dish due to extensive domain-specific knowledge. Internal context such as attention, motivation and emotions can modulate their acuity. In turn, the external context, e.g., whether they are judging in a cooking show vs. enjoying dinner with family, can modulate sensory acuity by modulating internal context.

**Fig. 1:**
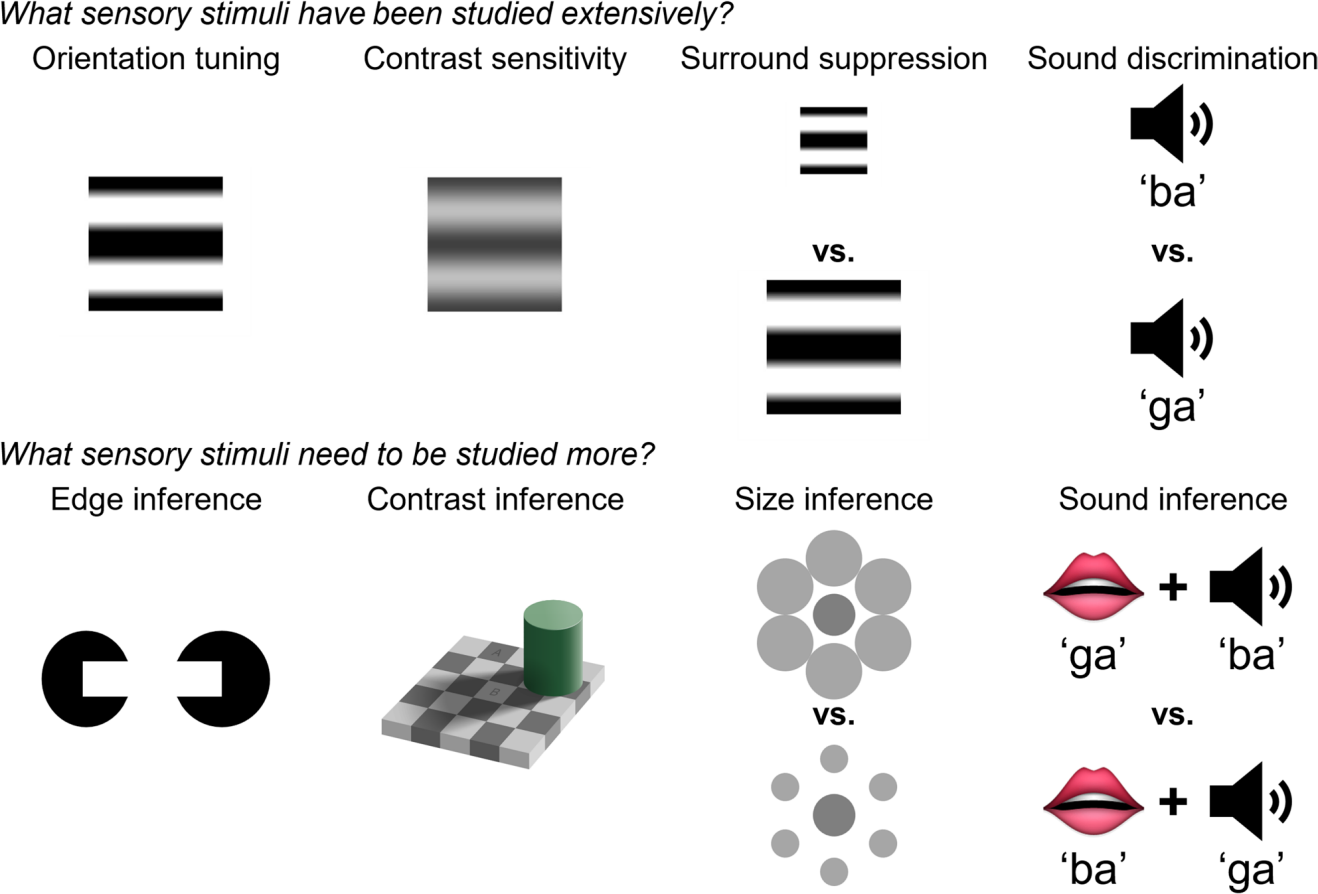
To study the neural code of perceptual inference, we need to utilize sensory stimuli whose perception is shaped by prior expectations. *Top*: Sensory stimuli conventionally used in neuroscience experiments. These stimuli are useful for studying sensory fidelity. From *left* to *right*: a horizontal static grating with 100% contrast; a horizontal static grating with 25% contrast; two horizontal static gratings with different sizes; and auditory discrimination between ‘ba’ and ‘ga’. *Bottom*: For these sensory stimuli, neural representation that is faithful to sensory information can be dissociated from the representation of perceptual inference. These stimuli are useful for studying various inference problems. From *left* to *right*: illusory contours for inferring edges [13]; checker shadow illusion for inferring contrast [14]; Ebbinghaus illusion for inferring size [15]; and McGurk effect for inferring sound based on audio-visual multisensory inputs [16]

In many experimental settings, the neural code that optimizes perceptual inference is indistinct from the code that optimizes sensory fidelity. These two codes are identical when the prior probability is uniform across the range of alternative perceptual interpretations in the experimental context. To further our understanding of the neural mechanisms underlying perceptual inference, we need more experiments that impose strong non-uniform priors and ambiguous sensory inputs (Fig. 1).

## Discrimination-generalization tradeoff

Let us imagine a ‘perceptual space’, where similar concepts are embedded near each other [17]. These ‘concepts’ are hypotheses, or plausible interpretations of sensory inputs. Both prior expectations and posterior percepts can be mapped onto the perceptual space. Concepts in the perceptual space can be viewed as categories; the boundaries demarcate the distinction between nearby categories (similar concepts).

The neural representation of a sensory input designates a probability distribution in the perceptual space. Representational overlap indicates multiple perceptual interpretations for a given neural activity pattern in the overlapping zone. Neural activity patterns in the overlapping zone can either be discriminated into different categories or generalized into the same category. Further, representational overlap implies redundancy between neurons encoding these representations, indicating lower efficiency and higher robustness. Thus, representational overlap in the perceptual space mediates both the tradeoff between discriminability vs. generalizability, as well as the tradeoff between efficiency vs. robustness.

Discrimination vs. generalization tradeoff is inherent to any categorization problem [18]. For example, if you find yourself lost at night with no way to call for help, you would focus on detecting a human shape. This may lead you to generalize any plausible shape into a human being. However, if you’re worried about vicious wild animals in the area, you would focus more on differentiating a human shape from other animal shapes. This would bias your brain to discriminate shapes rather than generalize them. Thus, in a neural code for inference, the inference problem at hand can flexibly shift the boundaries between perceptual categories. In contrast, a neural code for sensory fidelity has fixed boundaries between perceptual categories.

**Fig. 2:**
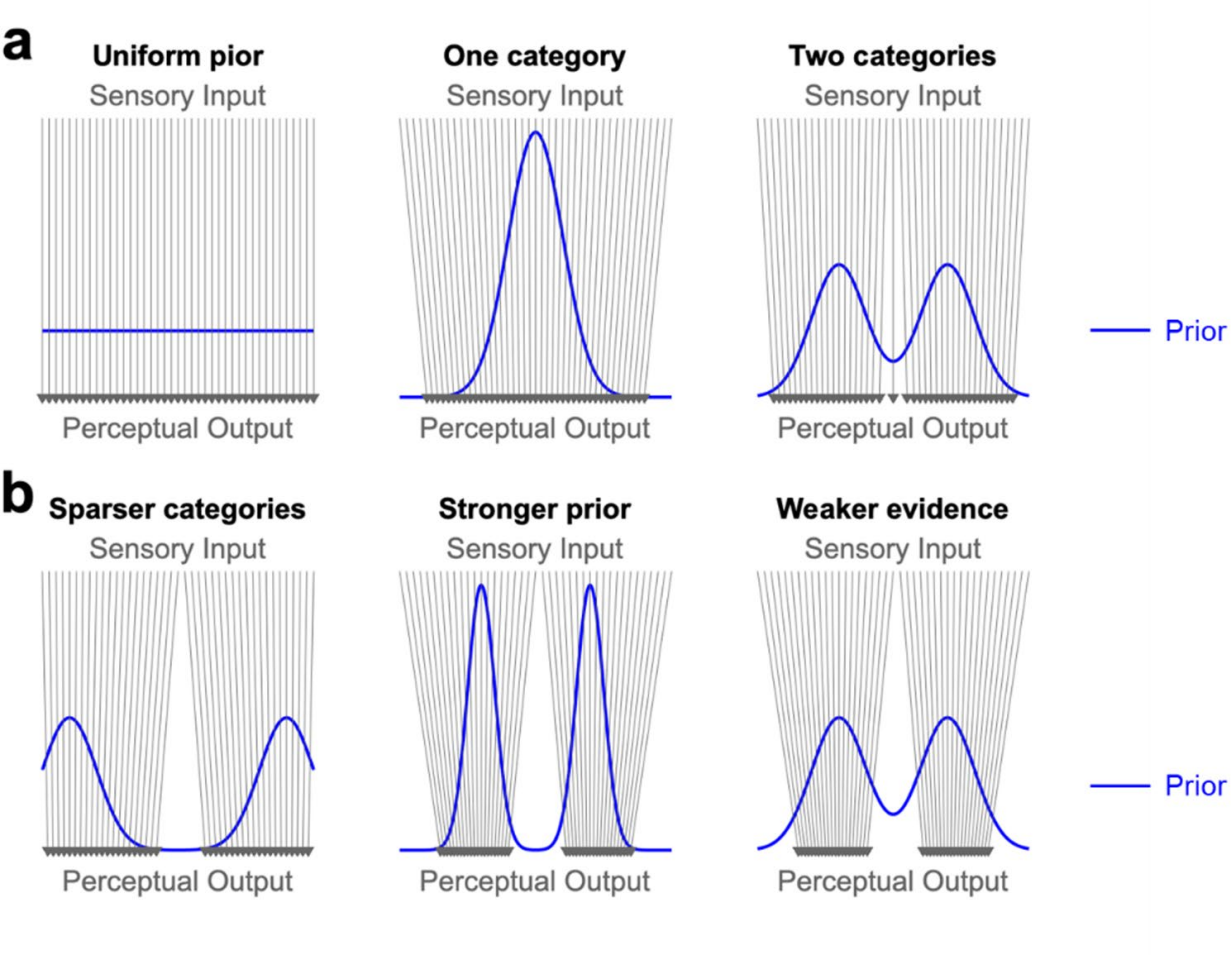
Perceptual magnet effect: discrete categories in the perceptual space act as magnets, pulling nearby sensory inputs towards the mode of each category [19]. *Blue lines* denote prior distributions; *gray arrows* denote the transformation from sensory inputs to perceptual outputs. **a** Perceptual outputs are warped relative to sensory inputs when the prior space is uneven. **b** Perceptual magnet effect is greater when adjacent perceptual categories are less similar (i.e., when categories are sparsely spaced in the perceptual space; *left*); when prior expectations for these categories are stronger (*center*); and when sensory inputs are weaker (*right*). Note the greater distortion from sensory input to perceptual output compared to the “Two categories” case in **a** *right*

The “perceptual magnet effect” arises as a consequence of inferring discrete perceptual categories from continuous sensory inputs, and predicts the optimal boundary between discrimination and generalization [19] (Fig. 2). The perceptual magnet effect is best known for phonetic categories (e.g., /i/ vs. /e/), where ambiguous in-between sounds are perceived as one of two phonemes. In an inference problem with an uneven prior space, the peaks in the prior space (i.e., the modes of each category) can act as attractors in the perceptual space. That is, sensory inputs get pulled into the nearest category, and the distortion from input to output is greatest when the sensory input is near the boundary of adjacent categories.

Both discrimination and generalization are crucial for rational and adaptive behavior. However, information theoretic metrics used to assess the neural code are often biased towards measuring the capacity for discrimination over generalization. Discriminability index, which is closely related to Fisher information, is an example of such a metric [1, 3]. Similarly, in neural decoding analyses, the cross-validation accuracy measures the classification performance.

More recently, decoding approaches have been modified to quantify generalization performance. For example, “inference decoding” aims to determine whether a neural population can generalize illusory contour representations to real edge representations, and vice versa [20]. Relatedly, “cross-condition generalization performance (CCGP)” analysis modifies conventional neural decoding such that the training set consists of trials from one group of conditions, while the test set consists of trials from a disjoint group of conditions [21]. CCGP aims to determine whether a set of rules in one context can be generalized to another context. An analogous approach has been used to assess invariant representations of object identity [22]. These approaches can be leveraged to assess the neural code’s capacity for generalization.

In addition, representational similarity analysis (RSA) provides a comprehensive characterization of the neural representation of perceptual space [23]. RSA can easily be modified to incorporate trial-to-trial reliability [24, 25]. RSA quantifies the similarity between representations of all pairs of perceptual categories. High similarity is indicative of closer distance in the perceptual space. As such, RSA offers a glimpse into the relative positions of the perceptual categories. Thus, rather than offering a one-sided assessment of the capacity for discrimination or generalization, RSA offers a way to probe the perceptual boundary between discrimination vs. generalization.

## Spatiotemporal axes of the neural code

**Fig. 3:**
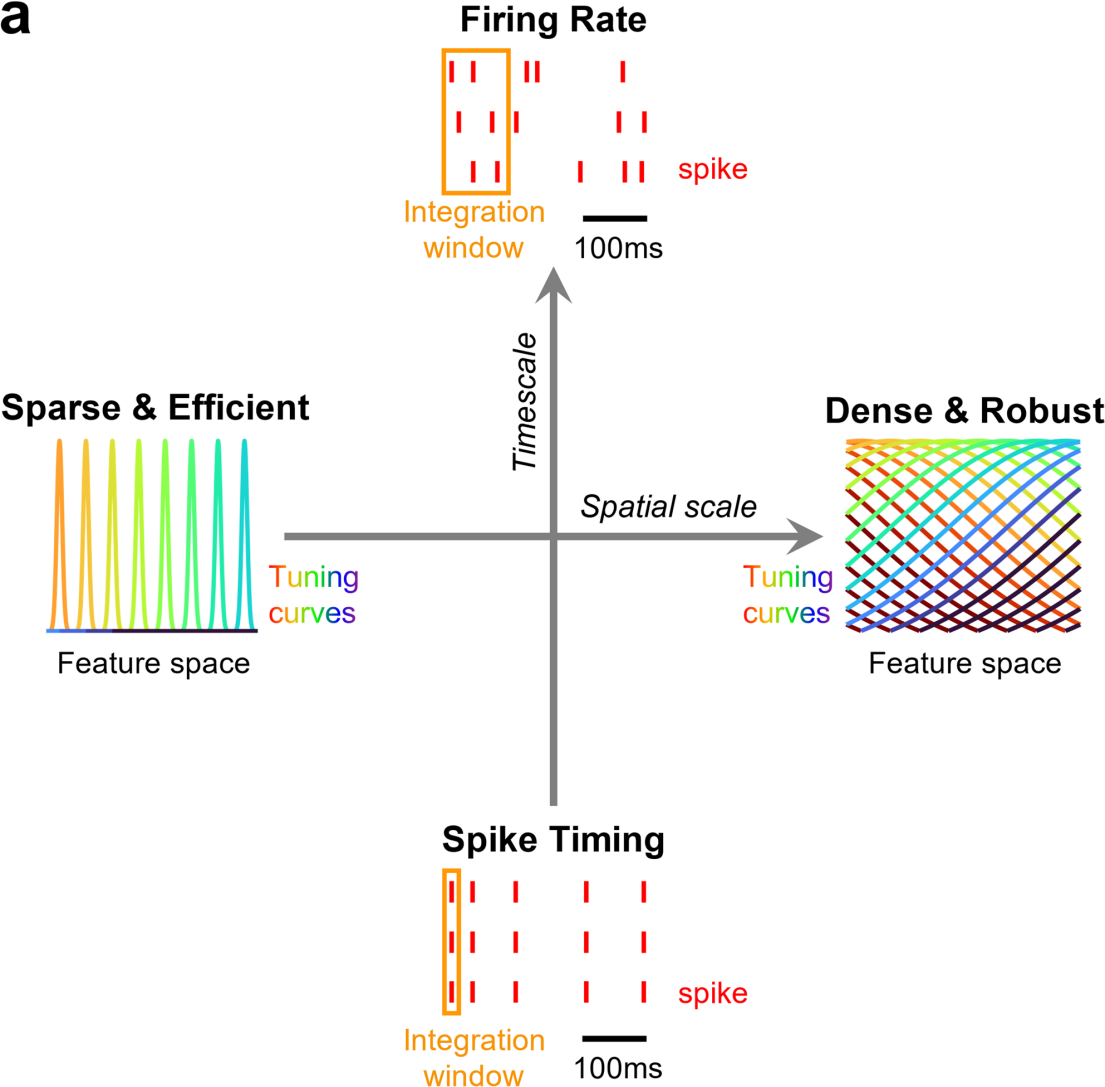
Spatiotemporal scale of the neural code for perception. **a** The spatial scale (*x-axis*) ranges from a sparse and efficient code (*left*) to a dense and robust code (*right*). Each colored curve depicts the tuning curve of a neuron in a hypothetical feature space. Tuning curves have increasing overlap with increasing spatial scale. The timescale (*y-axis*) ranges from a spike timing code (*bottom*) to a firing rate code (*top*). In the schematic, each row corresponds to a neuron; *red ticks* depict spikes, and *orange rectangles* depict the integration time window in a downstream area. In a spike timing code, the integration time window is thought to be on the order of milliseconds. In a firing rate code, the integration time window is on the order of tens to hundreds of milliseconds

 According to Marr’s three levels of analysis [26], understanding the neural code of perception entails elucidating the neural mechanisms at the computational, algorithmic and implementation levels. The core question at the computational level is concerned with what the goal of the computation is; the algorithmic level is concerned with how the computation is achieved step-by-step; and the implementation level is concerned with identifying the neural circuits that carry out each step of the computation. Discrimination vs. generalization describes a tradeoff at the computational level. At the algorithmic level, the tradeoffs to consider are the spatial scale and the timescale (Fig. 3).

The spatial scale is defined by the fraction of neurons participating in the neural representation of a percept. The spatial scale determines the efficiency vs. robustness tradeoff [25, 27]. In an efficient code, neural information per neuron is maximized such that each neuron encodes unique sensory information. An efficient code features highly selective neurons with sharp and non-overlapping tuning curves. As such, each sensory stimulus drives a sparse set of neurons. The infamous “grandmother cell”, i.e., a hypothetical cell that responds only to your grandmother, is an example of a sparse and efficient code [28, 29]. Conversely, in a robust code, information is redundant across neurons. Thus, each sensory stimulus drives a dense pattern of population activity. An example of a dense and robust neural code is the “population voting” scheme hypothesized for representing movement direction in motor cortex [30] and superior colliculus [31].

Efficient vs. robust neural codes each have their pros and cons. An efficient code can encode more information with the same number of neurons, all else being equal. On the other hand, a robust code is more resilient to neuronal damage. Here we are employing a narrow definition of robustness that entails redundancy but not reliability. Both efficiency and robustness can go up or down with trial-to-trial reliability. Thus, efficiency and robustness exist in a tradeoff but are not strictly zero-sum. As such, efficiency and robustness must be evaluated together for a comprehensive understanding of the neural code [25].

The spatial scale of the neural code is determined by the dimensionality of the perceptual space. Thus, to determine the spatial scale, we need to know the transformation between the neural state space and the perceptual space. The neural state space is the N-dimensional space of N neurons where each axis denotes the activity of each neuron. Perceptual differences are always relevant to neural activity, but neural activity differences are sometimes irrelevant to perception. As such, the perceptual space is a subspace of the neural state space. Thus, the dimensionality of the neural state space sets the upper bound of the dimensionality of the perceptual space, hence the upper bound of the spatial scale.

Relatedly, a popular method for assessing coding efficiency is to determine the saturation point for neural information as a function of the number of neurons [3, 4]. Higher number of neurons at the saturation point indicates lower redundancy and higher efficiency. On the other hand, robustness can be assessed by the consistency of representations between distinct sets of neurons [25, 32].

The tradeoff between efficiency vs. robustness and discrimination vs. generalization can be controlled via independent mechanisms, but they can also be mechanistically linked [33]. For instance, the density and evenness of the prior space are related to both tradeoffs (Fig. 2). If the perceptual categories are densely and robustly encoded in the prior space, the prior space becomes approximately uniform (Fig. 2a *left*). In this scenario, the distortion from sensory input to perceptual output is minimal. Here, two adjacent stimuli are more likely to be discriminated than lumped together. Conversely, if the perceptual categories are sparsely and efficiently encoded in the prior space, generalization becomes more prevalent (Fig. 2b). In sum, dense and robust encoding of categories in the prior space is favorable for discrimination, whereas sparse and efficient encoding of categories in the prior space is favorable for generalization.

The timescale of the neural code is an equally important algorithmic axis of the neural code. The timescale is defined as the time window of neural activity relevant for the perceptual readout. The axis of timescale is conceptually orthogonal to the axis of spatial scale in that any arbitrary combination of timescale and spatial scale is conceivable (Fig. 3). However, the problem of timescale is inseparable from the problem of spatial scale when interpreting the neural code: If one is misconstrued, the other is highly likely to also be misconstrued. The timescale of the neural code is debated between millisecond timescale to hundreds of milliseconds [34–38]. A spike timing code refers to a neural code where information is integrated over ~ 10^0^ to ~ 10^1^ ms timescale [39, 40]. In a spike timing code, neural ensembles must coordinate with millisecond synchrony to relay information downstream [37, 38]. On the other hand, a firing rate code has integration window of ~ 10^1^ to ~ 10^2^ ms timescale. In a firing rate code, neural information is determined by the neural vector of spike counts within the integration window [35, 36].

The timescale of the neural code is debated to this day [41–46]. On one extreme of this debate, higher firing rate is meaningless unless there is excess synchrony across neurons. On the other extreme, synchrony adds no information when spike counts within the integration window are the same. In practice, the timescale likely forms a gradient such that the downstream impact is maximal when neurons are coordinated at millisecond timescale, and has diminishing but non-negligible impact with longer timescales.

As with the spatial scale, accurate determination of the timescale requires knowledge of the transformation between neural state space and perceptual space. However, analyzing neural data with different time windows starting at sensory onset can offer insights into the question of timescale. For example, neural information, or decoding performance, will increase then saturate with longer time windows; the saturation point suggests the relevant timescale. In this vein, the time window that exhibits peak representational consistency between distinct neuronal subsets also hints at the relevant timescale.

An intriguing possibility is that multiple timescales are multiplexed such that distinct information are processed at distinct timescales [47]. For example, feedforward processing may rely on a fast spike timing code, and feedback processing on a slower firing rate code. In support, many studies have reported precise spike timing and millisecond synchrony for feedforward sensory processing, but slower and less precise rate increase for feedback processing [48, 49]. Relatedly, gamma rhythms (30–80 Hz) have been associated with spike synchrony and feedforward processing, whereas slower alpha (7–15 Hz) and beta rhythms (15–30 Hz) have been associated with top-down feedback processing [43, 50–53].

The timescale and spatial scale are distinct axes of the neural code, but a sparse and efficient code has typically been associated with a spike timing code and a dense and robust code with a firing rate code. Proponents of the efficient code argue that sparsity, whether it be spatial (i.e., fraction of active neurons at a time) or temporal (i.e., fraction of time that a neuron is active), is beneficial because it is energy efficient [54–59]. That is, given that generating an action potential consumes biological energy, it is most energy efficient to implement a neural code where information per action potential is maximized. On the other hand, a dense population code is characterized by broad overlapping tuning curves. In other words, each neuron responds to a range of stimuli in a graded fashion, with peak response to its most preferred stimulus and gradually decreasing responses to adjacent stimuli. Tuning curves show such smoothly varying responses when plotted with firing rate as the y-axis. Thus, dense population codes are often associated with a firing rate code, and sparse efficient codes with a spike timing code.

In sum, we discussed three axes of the neural code: discriminability vs. generalizability, efficiency vs. robustness, and synchrony vs. rate. Although these axes highlight the tradeoffs, all aspects of the neural code are affected by the reliability of neural representations because low reliability degrades the neural code. Further, we discussed metrics for evaluating these axes. We discussed information metrics, discriminability index and cross-validated decoding accuracy for assessing discriminability; inference decoding and CCGP for assessing generalizability; and RSA for assessing both discriminability and generalizability. We suggested that the neural dimensionality is indicative of the position along the efficiency vs. robustness axis; the number of neurons at which information or decoding performance saturates is indicative of efficiency; and the representational consistency is indicative of robustness. In addition, we suggested that analyzing the time window from sensory onset at which neural information, decoding performance, and representational consistency is maximized may indicate the relevant timescale. Together, these diverse metrics enable holistic evaluation of the neural code.

## Neural implementation of perceptual inference

So far, we have considered the neural computations and algorithms underlying perceptual inference. In this section, we discuss the neural implementation of perceptual inference. As with the computational and algorithmic levels, details at the implementation level are critical for correctly interpreting the neural code.

How is the Bayesian likelihood, prior and posterior implemented in the brain [10, 60]? To study how sensory inputs are transformed to perceptual outputs, it is desirable to study situations where there are experimentally measurable differences between sensory inputs and perceptual outputs. Perceptual outputs diverge from sensory inputs when there is a strong prior expectation about the sensory stimulus that is different from the stimulus itself. Illusions are a prime example where the difference between sensory inputs and perceptual outputs can be measured with psychophysics experiments. In the Bayesian framework, illusions can be interpreted as rational mistakes in perception that occur when sensory evidence does not match the prior, and the mismatching prior is strong enough to bias perception away from the sensory evidence (Fig. 1*bottom*). Thus, illusions can be leveraged to probe the brain’s prior model of the sensory world [61]. Further, we can study the neural implementation of inference by deciphering where and how sensory inputs become integrated with prior expectations. Following this logic, a recent study identified layer 2/3 of primary visual cortex (V1) as the lowest region in the mouse visual hierarchy where illusory contour inference is encoded [20]. Further, this study found that pattern completion in V1 layer 2/3 mediates illusory contour encoding [20], which in turn is driven by top-down feedback inputs [62, 63]. Therefore, this study demonstrates that pattern completion in V1 layer 2/3 underlies the generalization of illusory contours to real contours. More broadly, pattern separation and pattern completion can serve as the neural mechanism for discrimination and generalization, respectively [64].

How is the likelihood implemented? The brain can employ either the modular scheme or the transform scheme when implementing Bayesian inference [10]. According to the modular scheme, the likelihood, the prior and the posterior are encoded by distinct neural populations. Thus, the modular scheme predicts that there is a neural population dedicated to representing the likelihood. A typical implementation of the modular scheme would have the likelihood be represented in lower sensory areas [60, 65, 66], and the posterior be represented in a higher sensory area or an association area [67]. The alternative is the transform scheme, where the prior and/or the likelihood is encoded as a latent variable (e.g., synaptic weights), rather than be explicitly represented by the activity pattern of a neural population [10]. We will elaborate on these implementations and their implications in the section below, when we discuss the sensory hierarchy.

How is the prior implemented? To decipher the neural implementation of the prior, it is useful to divide the prior into two components: stable and dynamic. The stable prior is learned through experience. For example, illusory contours emerge from a lifetime’s experience of seeing edges. Another example where a stable, learned prior influences perception is the perceptual magnet effect, described above[19]. Learning discrete perceptual categories in a continuous sensory feature space leads to distorted sensory representations at the neural level, and the perceptual magnet effect at the perceptual level [65, 68, 69].

In comparison, the dynamic prior is modulated by the subject’s internal state and external context. For example, in a cued attention task, the cue dynamically adjusts the subject’s expectations about the upcoming sensory stimulus [70]. Alternatively, the probability that each stimulus will appear can be implicitly modulated in a block structure [71, 73]. A recent study showed that in a visual detection task where the probability of the visual stimulus appearing on the left vs. right was modulated in an implicit block structure, mice behaved according to a dynamically updated prior that approximated Bayesian optimality. Further, they found that this behaviorally relevant prior information was represented brain-wide in the pre-stimulus period, from thalamus to sensory, association and motor cortices [73].

The stable prior can be dynamically modulated. Recent work has demonstrated that mice trained on a visual orientation discrimination task show warped representations of orientation in primary visual cortex [68, 69], consistent with the perceptual magnet effect. This distortion appeared post-training in the context of the orientation discrimination task, even without explicit manipulation of prior expectations. Interestingly, the representations were not distorted when the trained mice passively viewed the same visual stimuli outside of the task context, indicating that the learned prior was dynamically modulated by the context [69]. Thus, the animal’s motivation for reward can dynamically influence the sensory prior in a sensory-reward association paradigm. Another prominent example where the perceptual magnet effect is dynamically modulated is the McGurk effect [16] (Fig. 1). In this case, ‘ba’ and ‘ga’ are phoneme categories that act as “perceptual magnets”, and the “magnetic pull” is determined by the visual input, i.e., the video of a person enunciating these phonemes.

As illustrated by the perceptual magnet effect, the transformation from continuous sensory feature space to discrete perceptual categories is mediated by uneven priors. The distortion from sensory input to perceptual output is greater when the sensory evidence is weaker, i.e., more ambiguous; and when the prior space is more uneven, i.e., when the bumps corresponding to perceptual categories are tall and sparsely spaced (Fig. 2b). In other words, these are the conditions in which the code for sensory fidelity is most distinct from the code for perceptual inference.

The stable and dynamic components of the prior have distinct implementations. Since a stable prior is learned through sensory experience, it can be embedded in neural connectivity through synaptic plasticity. For example, illusory contour perception arises from a strong prior for continuous edges [20], which can be implemented by the like-to-like connectivity of co-tuned neurons [74–77]. On the other hand, dynamic priors can be implemented by ongoing neural activity before the sensory stimulus [67, 73, 78].

How is the posterior implemented? It is debated whether neural activity on single trials represents the entire posterior probability distribution as a probabilistic population code [6, 79], or whether single trials represent individual samples of the posterior probability distribution [9, 80]. In the latter scenario, trial-to-trial variability encodes stimulus uncertainty as a direct consequence of probabilistic inference.

In sum, we have discussed how the likelihood, the prior, and the posterior may be implemented in the brain. To decipher the implementation, we need experiments that allow us to distinguish the representation of sensory inputs from the representation of perception. This distinction would allow us to identify brain areas that represent the sensory evidence, the likelihood, and the posterior. Subsequently, we would be able to interrogate how the transformation happens. To this end, we can leverage strong priors that are either learned (static priors), or temporarily imposed (dynamics priors). Examples of static priors pulling perception away from sensory inputs are illustrated in Fig. 1. Examples of dynamic priors include cued attention tasks, dynamic foraging tasks, and reversal tasks. Further, we can leverage neural manipulations to investigate how neural networks transform the likelihood to the posterior, i.e., the neural mechanisms of Bayesian inference.

## Neural mechanisms of perceptual inference across the sensory hierarchy

An important consideration when deciphering the neural mechanism of perceptual inference across the sensory hierarchy is that the computational, algorithmic and implementation level mechanisms all constrain one another. Ideally, we would need to address Marr’s three levels in parallel to gain a comprehensive understanding of the brain mechanism for perceptual inference.

**Fig. 4:**
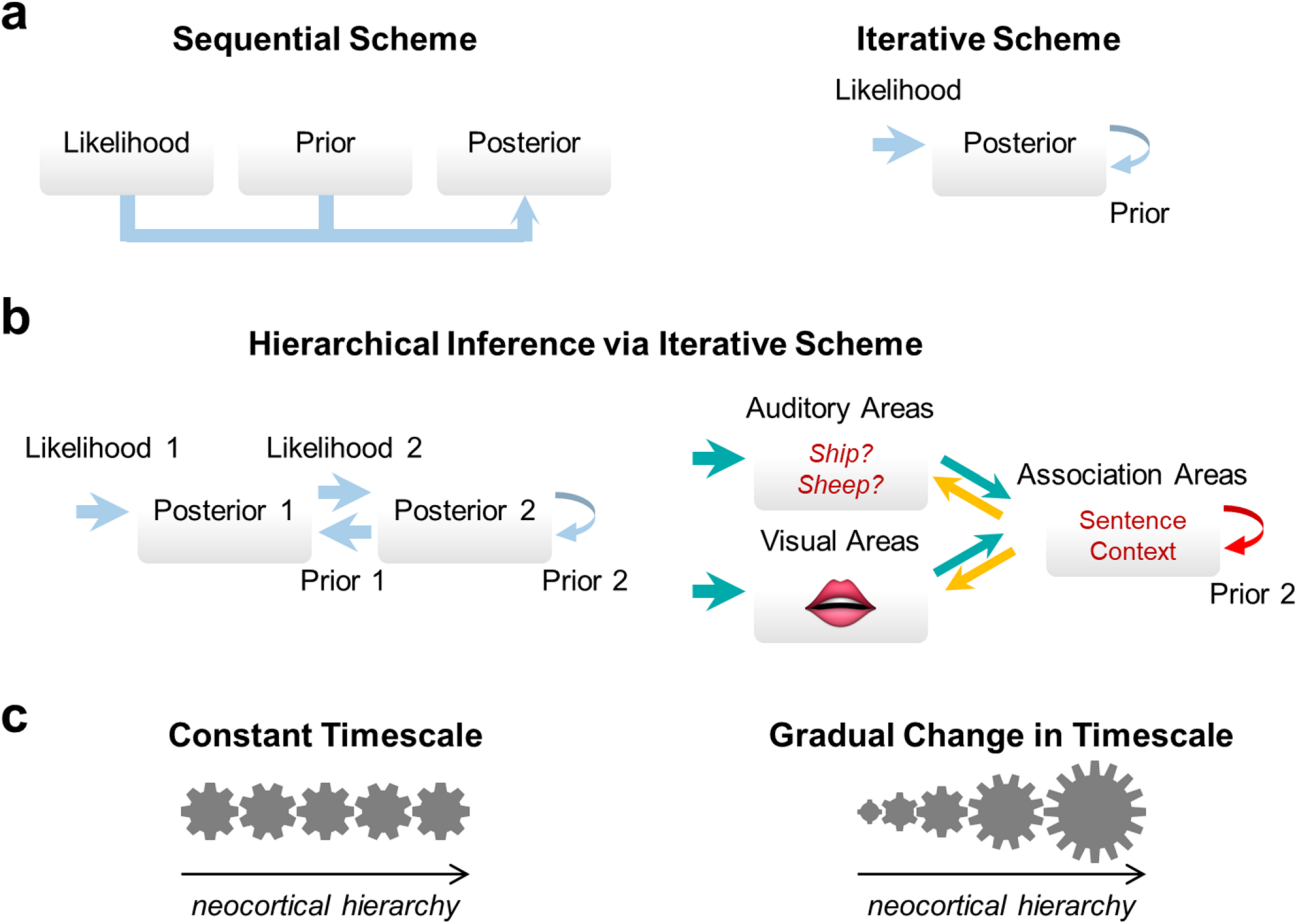
Perceptual inference mechanisms across the sensory hierarchy. **a-b** Possible implementations across the sensory hierarchy are depicted. **a** The posterior of Bayesian inference can be computed sequentially or iteratively. **b** In hierarchical inference, the posterior at each level of inference must be inferred in a cohesive manner (*left*). For example, whether you heard ‘ship’ or ‘sheep’ is a triple inference problem, requiring auditory inference of the sound, visual inference of the speaker’s lip movement, and higher-level cognitive inference of the word in the sentence (*right*; feedforward pathway in *teal*, feedback pathway in *orange* and recurrent pathway in *red*). **c** The spatiotemporal scale of the neural code may stay constant, or change across the hierarchy. When cogwheels are interlocked, the rate of each cogwheel spinning is inversely proportional to its diameter

To understand the computational level mechanism across the sensory hierarchy, we must determine whether perceptual inference is computed in a sequential manner, or an iterative manner (Fig. 4a). If the computation is sequential, it is possible to figure out the brain-wide mechanism by dissecting each area separately. If the computation is iterative, recurrent interactions across the hierarchy become critical. In either case, we need to elucidate the computation executed in each sensory area and then determine its algorithm and implementation. In the iterative scenario, we need to further elucidate the computations and algorithms implemented by feedforward and feedback connections between sensory areas.

The key to elucidating how perceptual inference is computed across the sensory hierarchy is to first determine the inference problem being solved by each brain region, then to determine whether that area is encoding the likelihood, the prior or the posterior of that inference problem. The brain may solve an inference problem by directly transforming the sensory input to the perceptual output. In this case, all engaged brain regions would be trying to solve the same inference problem. This can be implemented with either the sequential or the iterative scheme (Fig. 4a).

Alternatively, the brain may employ hierarchical inference, where the perceptual inference problem is broken down into lower- and higher-level subproblems. For example, when trying to discern whether one heard ‘ship’ or ‘sheep’ in a conversation, the lower-level inference problem is the phoneme (/i/ vs. /e/), and the higher-level inference problem is the meaning of the word in the sentence. The sensory cortical hierarchy can solve a hierarchical inference problem by having each area in the hierarchy solve its own inference problem [81–83]. A possible implementation is depicted in Fig. 4b, where feedforward activity encodes the likelihood and the feedback activity encodes the prior, and the posterior is iteratively updated through hierarchical processing. To solve a hierarchical inference problem, the entire hierarchy needs to converge to a globally coherent solution at all levels of inference. This process entails iterative refinement of posterior beliefs across all levels of the hierarchy during perceptual inference [83]. Thus, hierarchical inference is compatible with the iterative scheme, but not the sequential scheme.

Once we know which inference problem the brain area of interest is trying to solve, we can determine whether the neural representation in that area encodes the likelihood, the prior or the posterior. Here, we need to consider whether the implementation adheres to the modular view or the transform view, as described above [10]. The modular view is compatible with the sequential scheme and can be implemented by a feedforward network (Fig. 4a *left*). The transform view is compatible with both the sequential scheme and the iterative scheme.

The causal role of each brain area in the sensory hierarchy during perceptual inference has important implications for the mechanism. If perceptual inference is computed sequentially, a single brain region at the top of the sensory hierarchy (e.g., prefrontal cortex or posterior parietal cortex) would be necessary and sufficient for perception [83, 84]. This implies that feedforward connections are sufficient for implementing the sequential computations of perceptual inference, rendering feedback activity as mere correlates of perception. On the contrary, if perceptual inference is computed iteratively, the necessary and sufficient brain activity for perception would entail recurrent activity involving multiple levels of the sensory hierarchy [48, 85]. This implies that feedback activity in lower sensory areas does have a causal role for perception [20, 86, 87]. A middle ground for these opposing views would be that feedforward processing is sufficient for perception when sensory inputs are clear and unambiguous, but feedback activity is necessary when sensory inputs are incomplete or ambiguous. In the example of the ‘ship’ vs. ‘sheep’ inference problem, the iterative scheme, but not the sequential scheme, would predict that perturbing association cortex at the time of the sensory input would change the auditory and visual representations in sensory cortices. In other words, the feedback pathway has a causal role during perceptual inference in the iterative scheme, but not the sequential scheme, in Fig. 4b *right*.

Notably, whether inference is computed sequentially or iteratively (Fig. 4a), at a single level or at multiple hierarchical levels (Fig. 4b), and according to the modular or the transform view, may depend on the specific inference problem at hand. These schemes describe the computational level mechanisms of perceptual inference across the sensory hierarchy.

At the algorithmic level, we need to determine the spatiotemporal scale of the neural code in each area of the sensory hierarchy. Two questions are of particular interest. The first question asks whether the spatiotemporal scale stays constant across brain areas (Fig. 4c). If the scale does change, we need to determine whether the change is irregular or systematic across the hierarchy, although irregular change is unlikely due to a lack of supporting evidence. The second question asks whether feedforward and feedback communication from and to a brain region have matched spatial and/or temporal scales.

Recent literature suggested that the timescale of neural computation may lengthen progressively along the sensory hierarchy [88–90]. According to the sequential view, this result would simply imply that later stages of perceptual inference entail longer time constants of computation. On the contrary, the iterative view poses challenges for the implementation of incremental timescales across the hierarchy, as recurrent activity across the sensory hierarchy would need to resonate. To enable recurrent communication between multiple areas with different timescales, the sensory hierarchy machinery would need to function like cogwheels of different diameters interlocked with one another (Fig. 4c). Alternatively, the systematic increase in timescale may be limited to the feedforward communication during the initial sweep of feedforward activity; in this case, a fixed time constant for feedback communication can underlie global resonance.

The idea that the timescale changes across the sensory hierarchy is intriguing, but most theories of corticocortical communications are incompatible with this view [91]. For one, communication through coherence hypothesis [92, 93] presumes a spike timing code across the hierarchy and proposes that coherence between the phase of gamma oscillations (30–80 Hz) determines the effectiveness of corticocortical communication. In comparison, communication subspace hypothesis [94] proposes that the neural activity pattern in each cortical area determines the effectiveness of communication between these areas. The neural activity pattern is defined by the spike counts of neurons in each area during a fixed time window (100ms in the original paper [94]). Thus, the communication subspace hypothesis is based on the premise of a firing rate code with fixed integration window across the cortical hierarchy.

As with the timescale, the spatial scale of the neural code may stay constant or change across the sensory hierarchy. The receptive field size and object invariance increases across the hierarchy [95–98], suggesting that the neural code becomes systematically denser along the hierarchy. Then again, neurons are selective to increasingly complex features up the hierarchy, suggesting a systematic increase in coding sparsity [29, 95, 99]. These two gradients may be balanced such that the coding sparsity stays constant along the hierarchy [100].

An important consideration for the spatial scale of the neural code across the hierarchy is the conjunctive (AND) vs. disjunctive (OR) properties of feedforward connections. If feedforward connections tend to be conjunctive, neural representations would tend to grow sparser and more efficient across the hierarchy, and vice versa for disjunctive feedforward connections. If there is a systematic change in spatial scale, the gradient would depend on the degree to which feedforward connections are convergent vs. divergent [101, 102].

Yet another possibility is that the spatial scale changes during the timecourse of sensory processing. Lee and Mumford [83] proposed that during hierarchical inference, the brain generates multiple “hypothesis chains” upon sensory input. A hypothesis chain links hypotheses at every level of hierarchical inference and corresponds to one coherent percept. They posited that over the course of sensory processing, the most likely hypothesis chain is selected via iterative filtering. This would predict that neural representations should become sparser as sensory processing progresses. In addition, Lee and Mumford [83] posited that feedforward processing mediates hypothesis generation, and feedback processing mediates hypothesis selection. This would predict that feedforward processing is disjunctive, and feedback processing is conjunctive.

Taken together, the computational and algorithmic level mechanisms impose constraints on the implementation of perceptual inference across the sensory hierarchy. To elucidate the mechanism of perceptual across the hierarchy, we need to determine the role of each brain area and the pathways between them, in terms of the Bayesian prior, likelihood and posterior. Although the brain areas involved are likely to depend on the perceptual inference problem at hand, the transformation from the sensory input to the likelihood, and from the likelihood to the posterior may rely on common network mechanisms.

## Conclusion

Redefining the computational goal of perception from high-fidelity encoding of sensory information to rational perceptual inference calls for a reevaluation the computational, algorithmic and implementation level mechanisms of the neural code underlying perception (Table 1). The optimal neural code for fidelity vs. inference can be distinguished when strong prior expectations pull perceptual outputs away from sensory inputs, particularly when sensory evidence is weak or ambiguous. I propose that we need to leverage these situations to reexamine the optimal balance between the discrimination vs. generalization tradeoff, the efficiency vs. robustness tradeoff, and the spike timing vs. firing rate tradeoff. To evaluate these axes of the neural code, we need to determine the how, where, what and when of perceptual inference computation across the sensory hierarchy. To this end, we need to determine whether perceptual inference is computed in a sequential manner, or an iterative manner. Further, we need to determine whether the inference is solved in a hierarchical manner, and determine the inference problem pertaining to each brain area. Then we can determine whether the neural representation in each area encodes the prior, the likelihood or the posterior of that inference problem. Although the neural mechanism of perceptual inference may vary depending on the problem, these guidelines should facilitate our quest for cracking the neural code of perceptual inference. Once we elucidate the neural mechanisms of many different perceptual inference problems, we would be able to establish generalizable principles of the neural mechanism underlying perceptual inference.

**Table 1:** Marr’s three levels of analysis for perceptual inference

Computational	Sensory information → Perceptual inference Classification vs. Generalization
Algorithmic	Efficient sparse code vs. Robust population code Spike timing vs. Firing rate
Implementation	Which brain area(s) encode the prior, likelihood and posterior? Role of feedforward, feedback and recurrent communication Role of each cell type and layer

## Data Availability

No datasets were generated or analysed during the current study.
